# Supplementary material for: Splice-Junction-Based Mapping of Alternative Isoforms in the Human Proteome
Source: Cell Rep. Author manuscript; Available in PMC 2020 Jan 15. (PMC6961840; doi:10.1016/j.celrep.2019.11.026)
Supplement: 2 [file NIHMS1546469-supplement-2.zip › DF1/PXD010154/Esophagus/Esophagus_20_WDR47_NELEFTMQEAVQCLHALEEYCPSKDDYSK.pdf]

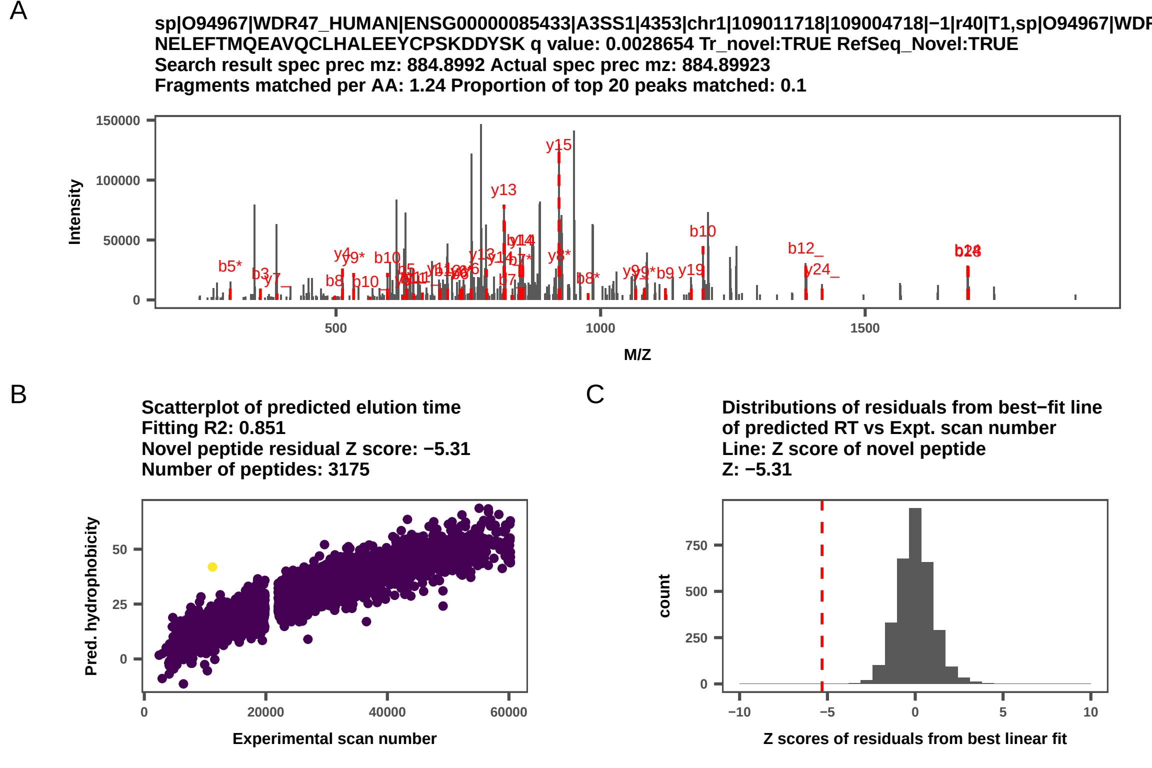
A
sp|O94967|WDR47_HUMAN|ENSG00000085433|A3SS1|4353|chr1|109011718|109004718|−1|r40|T1,sp|O94967|WDF
NELEFTMQEAVQCLHALEEYCPSKDDYSK q value: 0.0028654 Tr_novel:TRUE RefSeq_Novel:TRUE
Search result spec prec mz: 884.8992 Actual spec prec mz: 884.89923
Fragments matched per AA: 1.24 Proportion of top 20 peaks matched: 0.1
Intensity
150000
100000
50000
0
500
1000
1500
M/Z
y15
y13
b10
b12_
y24_
b5*
y19
B
Scatterplot of predicted elution time
Fitting R2: 0.851
Novel peptide residual Z score: −5.31
Number of peptides: 3175
Pred. hydrophobicity
50
25
0
0
20000
40000
60000
Experimental scan number
C
Distributions of residuals from best−fit line
of predicted RT vs Expt. scan number
Line: Z score of novel peptide
Z: −5.31
count
750
500
250
0
−10
−5
0
5
10
Z scores of residuals from best linear fit
